# Inflow/Outflow Boundary Conditions for Particle-Based Blood Flow Simulations: Application to Arterial Bifurcations and Trees

**DOI:** 10.1371/journal.pcbi.1004410

**Published:** 2015-08-28

**Authors:** Kirill Lykov, Xuejin Li, Huan Lei, Igor V. Pivkin, George Em Karniadakis

**Affiliations:** 1 Institute of Computational Science, Faculty of Informatics, University of Lugano, Lugano, Switzerland; 2 Division of Applied Mathematics, Brown University, Providence, Rhode Island, United States of America; 3 Pacific Northwest National Laboratory, Richland, Washington, United States of America,; 4 Swiss Institute of Bioinformatics, Lausanne, Switzerland; CANADA

## Abstract

When blood flows through a bifurcation, red blood cells (RBCs) travel into side branches at different hematocrit levels, and it is even possible that all RBCs enter into one branch only, leading to a complete separation of plasma and RBCs. To quantify this phenomenon via particle-based mesoscopic simulations, we developed a general framework for open boundary conditions in multiphase flows that is effective even for high hematocrit levels. The inflow at the inlet is duplicated from a fully developed flow generated in a pilot simulation with periodic boundary conditions. The outflow is controlled by adaptive forces to maintain the flow rate and velocity gradient at fixed values, while the particles leaving the arteriole at the outlet are removed from the system. Upon validation of this approach, we performed systematic 3D simulations to study plasma skimming in arterioles of diameters 20 to 32 microns. For a flow rate ratio 6:1 at the branches, we observed the “all-or-nothing” phenomenon with plasma only entering the low flow rate branch. We then simulated blood-plasma separation in arteriolar bifurcations with different bifurcation angles and same diameter of the daughter branches. Our simulations predict a significant increase in RBC flux through the main daughter branch as the bifurcation angle is increased. Finally, we demonstrated the effectiveness of the new methodology in simulations of blood flow in vessels with multiple inlets and outlets, constructed using an angiogenesis model.

## Author Summary

Blood tests, which provide a wealth of information on the state of human health, are often performed on cell-free samples. Therefore, blood-plasma separation needs to be achieved. A simple but effective solution for isolating plasma from blood utilizes capillary bifurcations. In a particle-based simulation study of plasma skimming in capillary bifurcations, the blood flow properties such as velocity and pressure fields differ drastically at the inlet and outlet regions. Therefore, a new open (non-periodic) boundary is required. In this paper, we have developed and validated a general parallel framework for open boundary conditions. This is a non-trivial enabling technology that could be used in all open boundary systems and all particle-based Lagrangian simulations. We performed systematic 3D simulations of blood flow in arteriolar bifurcations and elucidated the biophysical mechanism of blood-plasma separation as well as quantified the effects of branch size and bifurcation angle on cell separation efficiency, which have not been addressed before. We also demonstrated the applicability of the methodology in arterial trees with multiple inlets and outlets.

## Introduction

Blood is a biological fluid that delivers nutrients and oxygen to living cells and removes their waste products. The two major components of whole blood are red blood cells (RBCs) and plasma, *i*.*e*., RBCs constitute approximately 40% of the total blood volume, plasma around 55%, while the rest is taken up by white blood cells (WBCs) and platelets. Lateral migration of RBCs takes place in blood flow, leading to the formation of two phases, *i*.*e*., a core consisting mainly of RBCs and a cell-free plasma layer adjacent to the vessel wall where the platelets tend to concentrate [1, 2]. WBCs, which are larger and more rigid than RBCs, migrate toward the vessel wall through a process called margination [3]. The tendency of RBCs to concentrate at the vessel center also leads to plasma skimming—an asymmetric distribution of RBCs and plasma between two daughter branches when blood flows through a microvascular bifurcation. The RBCs prefer a daughter branch with higher flow rate leaving very few RBCs (even reaching zero) flowing into lower flow rate daughter branch [4].

Blood-plasma separation in bifurcations has been extensively investigated in the past few decades, and it is generally believed that three factors, feed hematocrit [5–7], size of parent channel [8, 9] and flow rate ratio of daughter branches [1], mainly determine the degree of plasma skimming that will occur [8]. Studies of blood flow through bifurcations have revealed significant variability for a complete RBC separation from the whole blood (*all-or-nothing* phenomenon). The theoretical critical flow rate ratio between the daughter branches for predicting such phenomenon is approximately 2.5:1 [5]. However, more recent experimental measurements showed that for this flow rate ratio only 88.7% of RBCs enter into the higher flow rate daughter branches [10]. This raises the question as to what ratio value is more meaningful in determining total blood-plasma separation.

Computational modeling and simulations can help us to investigate this issue. In past decades, numerical modeling of blood flow in capillaries has attracted increasing attention [11, 12]. For example, dynamic simulations can model how blood flow behaves in microfluidic channels [13–18] and predict human blood viscosity in silico [19]. Different cell models have also been employed for various qualitative and quantitative interpretations as well as predictions of biomechanical properties of RBCs with hematological diseases [20–23]. Examples include dynamic cell deformability for various stages of malaria-infected RBCs [24–28] and vaso-occlusion phenomena in sickle cell anemica [23]. However, most of these blood flow simulations were performed in systems with periodic boundary conditions (PBCs) along the flow direction, whereas very few studies so far have reported simulations of non-periodic flow [29–31]. In a previous study, we simulated the blood-plasma separation for healthy and diseased blood in microfluidic channels with geometrically symmetric bifurcation and confluence to satisfy the periodic flow assumption along the flow direction [32]. However, for a simulation study of plasma skimming in capillary bifurcations, the blood flow properties such as velocity and pressure fields differ drastically at the inlet and outlet regions. Therefore, the choice of PBCs is inappropriate for general cases, especially in arterial trees, and hence a new open (non-periodic) boundary is required; this is a non-trivial issue, especially for particle-based Lagrangian methods.

For an open boundary system, the velocity profile at the inlet is generally specified, whereas the outflow profiles are rarely known. For a single-phase system, the inflow condition could be simply obtained by extending the inflow length so that the flow becomes fully developed at the inlet. However, for multiphase systems the inflow conditions even for a fully developed flow are unknown—a situation similar to turbulent inflow in single phase. For example, for a blood flow, the flow and viscous properties as well as the cell-free layer (CFL) distribution in arteries differ greatly with change in hematocrit level and shear rate. Thus, the inflow length should be long enough to generate inflow condition for blood flow. As a consequence, it is totally inefficient and perhaps impossible to perform blood flow simulations using this “brute-force” approach because of prohibitively expensive computations. Recent works have focused on the development of new methods to solve these problems. For example, an attempt to develop new boundary conditions has been presented by Flekkoy *et al*. [33], in which the simulation domain includes an auxiliary buffer domain for particle generation. However, the complexity of the flux control makes it difficult to perform flow simulations. Recently, a new method for such open systems has been developed by Lei *et al*. [34], where they generated particles at the inlet according to the local flux and introduced adaptive forces to control the flow rate at the outlet. This method has been successfully applied to single phase flow in straight channels and in bifurcations [34]. However, in multiphase systems, e.g., flows with colloids, polymer chains or RBCs, it is difficult to insert them at the inlet and remove them at the outlet. Thus, existing methods cannot be readily extended to the cases of complex flows such as blood flow.

In this paper we present a general framework for open boundary systems including the inflow and outflow boundaries for particle-based approaches targeting simulations of multiphase flows. We implemented this framework in the context of parallel computations. We show that the particles flowing in a complex computational domain can be treated as a system in contact with a simpler subsystem with a fully developed flow for the inflow combined with an osmotic membrane to control the outflow.

## Methods

### Multiscale RBC model

We simulated the blood flow in arterioles with the help of a multiscale RBC (MS-RBC) model [35] based on the dissipative particle dynamics (DPD) approach [36–38]. For completeness, the MS-RBC model is briefly reviewed below, whereas details on the RBC model are available elsewhere [35, 39].

In the MS-RBC model, the cell membrane is modeled by a 2D triangulated network with *N*_*v*_ vertices connected by springs, where each vertex is represented by a DPD particle. The RBC membrane model takes into account the elastic energy, bending energy, and constraints of fixed surface area and enclosed volume, which is defined as

$$V=V_{s}+V_{b}+V_{a}+V_{v}$$ (1)

where *V*_*s*_ is the elastic energy that mimics the elastic spectrin network, given by

$$V_{s}=\sum_{i\in \text{springs}}[\frac{k_{B}Tl_{m}}{4p}\frac{3x_{i}^{2}-2x_{i}^{3}}{1-x_{i}}]+\sum_{\alpha \in \text{triangles}}\frac{1}{A_{\alpha }}[\frac{3\sqrt{3}k_{B}Tl_{m}^{3}x_{0}^{4}}{64p}\frac{4x_{0}^{2}-9x_{0}+6}{\left(1-x_{0}^{2}\right)}],$$ (2)

where *k*_*B*_ *T* is the energy unit, *A*_*α*_ is the area of triangle *α* formed by three vertices. Also, *x*_*i*_ = *l*_*i*_/*l*_*m*_, *x*_0_ = *l*_0_/*l*_*m*_, where *l*_*i*_ is the length of spring *i*, *l*_0_ and *l*_*m*_ are the equilibrium spring length and maximum spring extension, and *p* is the persistence length.

The cell membrane viscoelasticity is imposed by introducing a viscous force on each spring, which has the form,

$$F_{ij}^{D}=-\gamma^{T}v_{ij}-\gamma^{C}\left(v_{ij}\cdot e_{ij}\right)e_{ij},$$ (3)

$$F_{ij}^{R}dt=\sqrt{2k_{B}T}(\sqrt{2\gamma^{T}}d\bar{W_{ij}^{S}}+\sqrt{3\gamma^{C}-\gamma^{T}}\frac{tr[dW_{ij}]}{3}1)\cdot e_{ij},$$ (4)

where *γ* ^*T*^ and *γ* ^*C*^ are dissipative parameters; **v**_*ij*_ is the relative velocity of spring ends, and $d\bar{{\text{W}}_{ij}^{S}}=d{\text{W}}_{ij}^{S}-tr[d{\text{W}}_{ij}^{S}]\text{1}/3$ is the traceless symmetric part of a random matrix representing the Wiener increment.

The bending resistance of the RBC membrane is modeled by

$$V_{b}=\sum_{\alpha ,\beta \;\text{pair}}k_{b}[1-\cos(\theta_{\alpha \beta }-\theta_{0})],$$ (5)

where *k*_*b*_ is the bending modulus constant, *θ*_*αβ*_ is the instantaneous angle between two adjacent triangles having common edge, and *θ*_0_ is the spontaneous angle. In addition, the RBC model includes the area and volume conservation constraints, which mimic the area-incompressibility of the lipid bilayer and the incompressibility of the interior fluid, respectively. The corresponding energy terms are given by

$$V_{a}=\frac{k_{a}k_{B}T{\left(A-A_{0}\right)}^{2}}{2l_{0}^{2}A_{0}},\;V_{v}=\frac{k_{v}k_{B}T{\left(V-V_{0}\right)}^{2}}{2l_{0}^{3}V_{0}}$$ (6)

where *k*_*a*_ and *k*_*v*_ are the area and volume constraint coefficients. Here *A*_0_ and *V*_0_ are the equilibrium area and volume of a cell, respectively.

The MS-RBC model is multiscale, as the RBC can be represented on the spectrin level, where each spring in the network corresponds to a single spectrin tetramer with the equilibrium distance between two neighboring actin connections of ∼ 75 *nm*. On the other hand, for more efficient computations, the RBC network can also be highly coarse-grained with the equilibrium spring lengths of up to 500 ∼ 600 *nm*. In most simulations, we use *N*_*v*_ = 500, a highly coarse-grained RBC model which has been employed to conduct efficient simulations of RBCs in microcirculation [16, 35, 40, 41]. For comparison, we also consider two finer DPD cases with *N*_*v*_ = 2560 and *N*_*v*_ = 5000. The internal and external fluids are modeled by free DPD particles.

### Inflow and outflow boundary conditions

**Fig 1:**
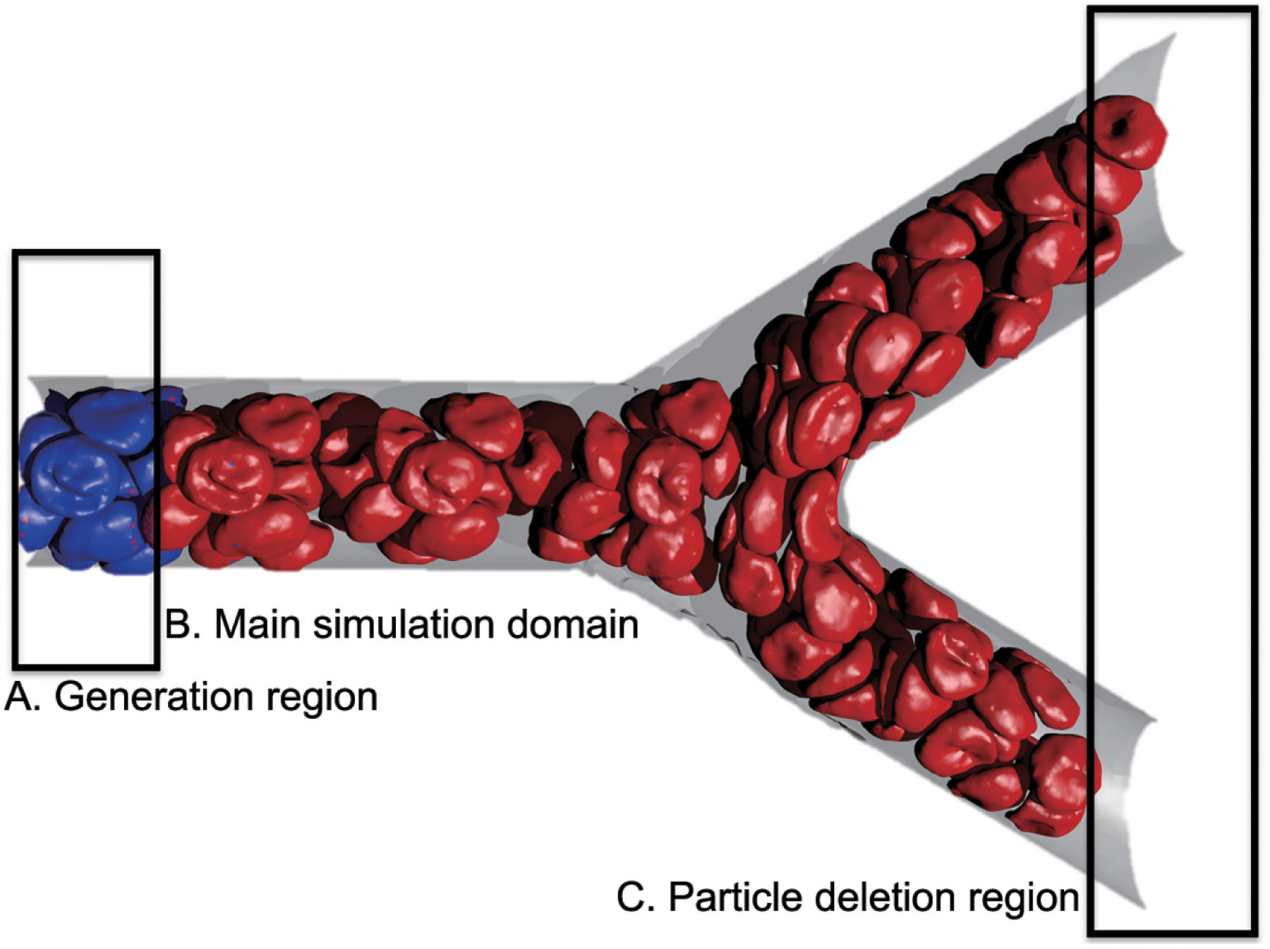
Simulation of blood flow in a microvascular bifurcation using the proposed open boundary conditions. The simulation domain is subdivided into three regions: **(A)** generating region with fully developed flow; **(B)** the main simulation domain; **(C)** the outlet region where particles are deleted. Fluid particles and frozen wall particles are not shown for clarity.

For simple computational domains where PBCs can be applied, our framework is shown to exhibit exactly the same flow characteristics as those obtained by imposing PBCs. Of course, what is more important is that it can be applied to domains where PBCs can not be employed. Unlike previous works, the proposed approach has a great advantage of being applicable to both simple fluid flows and suspensions. In the proposed scheme, the computational domain is divided into three regions as illustrated in Fig 1. Here, the simulation is performed in region B, while regions A and C are auxiliary. In order to generate an inflow in the main computational domain, we use the generating region as a source of new particles. At the same time, when a particle leaves the main simulation domain, it enters into the region C and is removed from the system.

**Fig 2:**
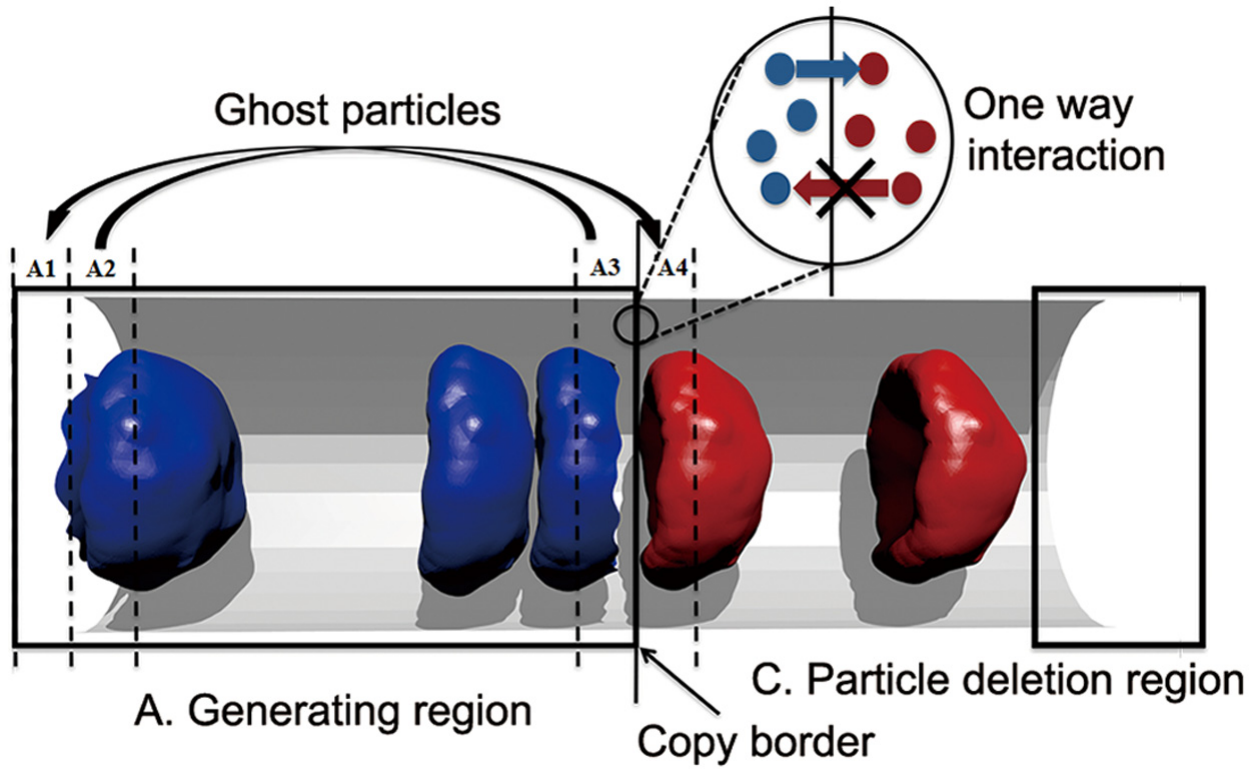
Schematic illustration of the computational domain of an open system. The generating region is divided into zones: zones A2 and A3 are sources of ghost particles while zones A1 and A4 are used for placing ghost particles. As soon as a particle crosses the copy border, its copy is created. There is one way interaction between particles in the generating region and particles in the main simulation domain.

To illustrate the approach in detail, let us consider a microtube flow of RBCs and plasma suspension as shown in Fig 2. In order to have a fully developed inflow in the main computational domain, the generating region is expected to mimic the flow in an infinite tube and be independent of the simulation in the main simulation domain. To reach these requirements, the PBCs in the generating region were implemented by ghost particles along with the particles shifting from one side of the domain to another side when they leave the periodic box. For all particles from zone A2 we create ghost particles and place them in zone A4, and a similar procedure is used for zones A3 and A1. The width of zones A1-A4 is the maximum of the cutoff radii of the force interaction used in simulations. Ghost particles are created after integration in the velocity-Verlet algorithm, but before computer processors exchange forces. The independence of the generating region from the main simulation domain is achieved by turning off forces acting from particles in the main simulation domain on the particles in the generating region. At the same time, the interactions in the opposite direction are preserved because it is desirable to prevent penetration of created particles and their topological structures into the generating region. To connect the aforementioned regions, we design a procedure to duplicate particles from the generating region to the main simulation domain. That is, when a particle in the generating region crosses the copy border, a duplication of the particle is created in the main simulation domain. This duplicated particle is created in the main simulation domain, and hence the inflow is fully developed.

The extension of the proposed inflow boundary conditions for blood flow requires extra care for the cell topology. Specifically, for the ghost interaction implementation, a ghost particle corresponding to a cell vertex needs to keep bonded potential terms such as bonds, angles, and dihedral angles. In addition, the size effect of the periodic domain for computing the corresponding interactions needs to be considered. The procedure of RBC generation at the inlet is also different from the one for a single particle. First, the RBC cannot be duplicated particle by particle, instead, the whole RBC is expected to be duplicated once its center-of-mass (COM) crosses the copy border between the generating domain and main simulation domain. Second, when a new RBC has been generated in the main simulation domain, some vertices of the new duplicated RBC may still be in the generating domain. Thus, we have to pay more attention to the RBC particles and their duplications in the generating domain because they may be in close contact and cause artificial strong repulsive interactions. To avoid this, we enforce the duplicated RBC to move exactly like the original RBC in the generating domain until the entire RBC lies fully inside the main simulation domain. Without extra effort, the proposed procedure allows us to add RBCs and fluid naturally to the main simulation domain. In particular, there are no artificial interactions because of the one way interaction between particles in the generating region and particles in the main simulation domain. It is worth mentioning that the proposed method can achieve a “seamless” connection between the generating region and main simulation domain, so the RBCs flowing in a complex computational domain can be treated as a system in contact with a simpler subsystem with a fully developed flow for the inflow. The periodic pattern observed in regions A2 and A3 is not unexpected since we run the simulation in this region periodically to generate the full developed inflow. An increase of the length of the generating region leads to a better time-averaged flow properties at the inlet and may eliminate or reduce such periodic pattern, but its effect to the simulation results is insignificant since the flow has already been fully developed at the inlet. Also, it requires extra computation time.

In order to impose the outflow boundary conditions for the simple fluid flow, we employ a method similar to Lei *et al*. [34]. Specifically, those particles leaving the simulation domain and entering into particle deletion region are reflected back to the main simulation domain with a probability (1 − *P*) depending on the particle number density *ρ* we want to preserve. They are computed at each iteration using the following algorithm:

1. Calculate density *ρ* in the main simulation domain.
2. Compute probability increment $dP=h*\frac{|\rho_{current}-\rho_{target}|}{\rho_{target}}$, where *h* is a weighting factor and it is set at 0.05 in this study.
3. If *ρ* ≤ *ρ*_*target*_, *P* is updated to (*P* + *dP*); otherwise it is *P* = (*P* − *dP*).
4. For a particle crossing the outflow plane, reflect the particle back with probability (1 − *P*).

We note that identical outflow boundary conditions might be implemented by applying adaptive forces in the vicinity of the outflow plane [34]; however, the formulation proposed here is much simpler to implement. A similar reflecting membrane has been used to generate a fluid flow in previous molecular dynamics simulations [42]. The outflow boundary conditions for RBCs are implemented in a different way. When the whole RBC is inside the region for cell deletion, we destroy the cell topology but leave particles in place and change their properties from the cell-like particles to fluid-like particles, which are removed downstream as described above. We found that this method outperforms an alternative implementation, where the whole cell is deleted because the removal may create density artifacts in the region of cell deletion.

## Results/Discussion

To validate the proposed open boundary conditions (OBCs), first a single phase flow (without RBCs) in straight microtubes is simulated and compared with an analytical solution. Numerical simulations are carried out in a 3D geometry representing the microtube used in DPD simulations. In all simulations, the solid walls are modeled by freezing layers of particles with bounce-back reflection to satisfy the no-slip boundary condition [43, 44]. Here, for simple fluid and, later, for plasma in blood suspension, the following DPD parameters are employed [34]: *a* = 4.0, *γ* = 30.0, *r*_*c*_ = 1.5, *k*_B_ *T* = 0.0945, *n* = 2.96. A generalized weight function, *w*(*r*) = (1 − *r*/*r*_*c*_)^*s*^, for dissipative force with *s* = 0.5 is also used in order to increase the viscosity of the DPD fluid [45, 46]. An external body force with magnitude of *g* = 0.1 is exerted on each fluid particle to generate a Poiseuille flow in the microtube. The microtube diameter is *d* = 10.0 *μm*.

**Fig 3:**
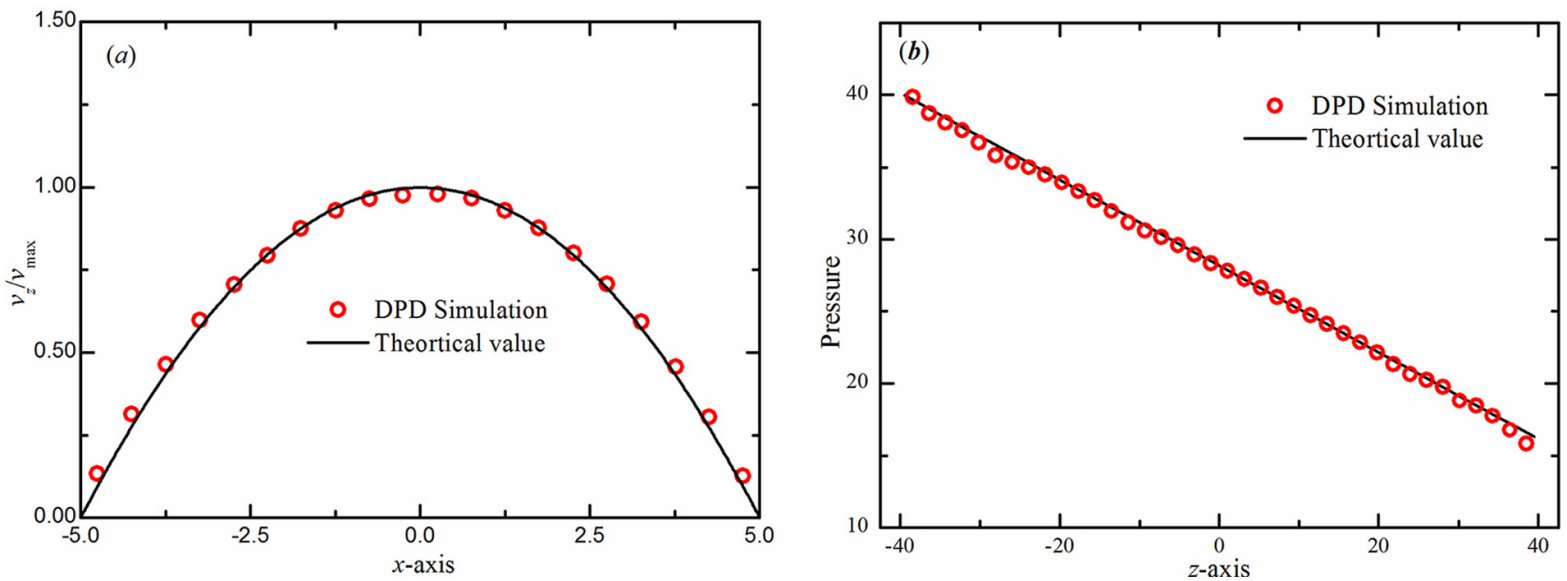
Verification of the accuracy of the proposed method. (***a***) Velocity profiles in the cross-flow (*x*-axis) direction of the Poiseuille flow corresponding to open boundary conditions. The incompressible Navier-Stokes solution is shown with lines. (***b***) Pressure profile for the Poiseuille flow along the flow (*z*-axis) direction. The symbols represent the DPD simulation results and the solid line represents the analytical solution.

Fig 3***a*** shows the average velocity profiles obtained from the simulations with the OBCs. The velocity profile is parabolic, which agrees well with the analytical prediction and proves the correctness of the scheme. For a more quantitative analysis, we compute the pressure profile along the flow (*z*-axis) direction (see Fig 3***b***). We find that the DPD simulation results are in good agreement with the analytical prediction given by *dP*/*dz* = 16*v*_*max*_ *η*/*d* ^2^ = *ng*, where *η* is the viscosity of the DPD fluid.

**Fig 4:**
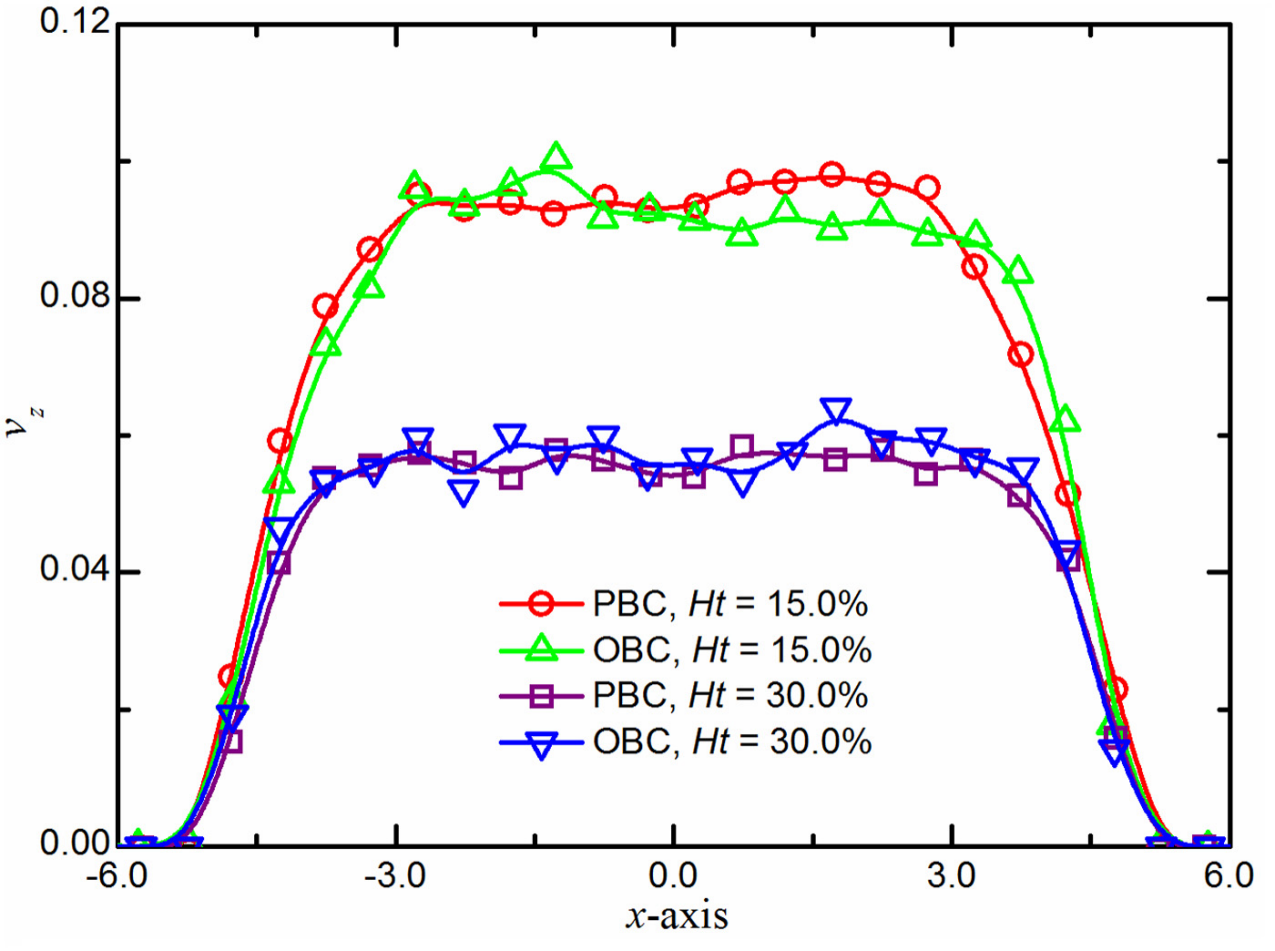
Validation of the open boundary conditions. Typical velocity profiles of blood flow in microtubes at *H*_*t*_ = 15.0% and 30.0%. The simulation results are compared to those in periodic systems at same hematocrit levels. *x* and *z* represent the radial and axial distances for the cylinder geometry; *v*_*z*_ is the velocity along the flow direction.

Having verified the single phase flow, we simulate the motion of RBC suspension through a straight tube. Fig 4 shows the average velocity profiles for blood flow at two different hematocrit levels (*H*_*t*_), *i*.*e*., *H*_*t*_ = 15.0% and 30.0%. In this plot, quasi-parabolic (or flat plug-like) shapes of the typical velocity profiles of blood flow are shown and compared against results obtained with PBCs. These results indicate that the blood flow in microtubes can be simply and accurately implemented by the described scheme.

**Fig 5:**
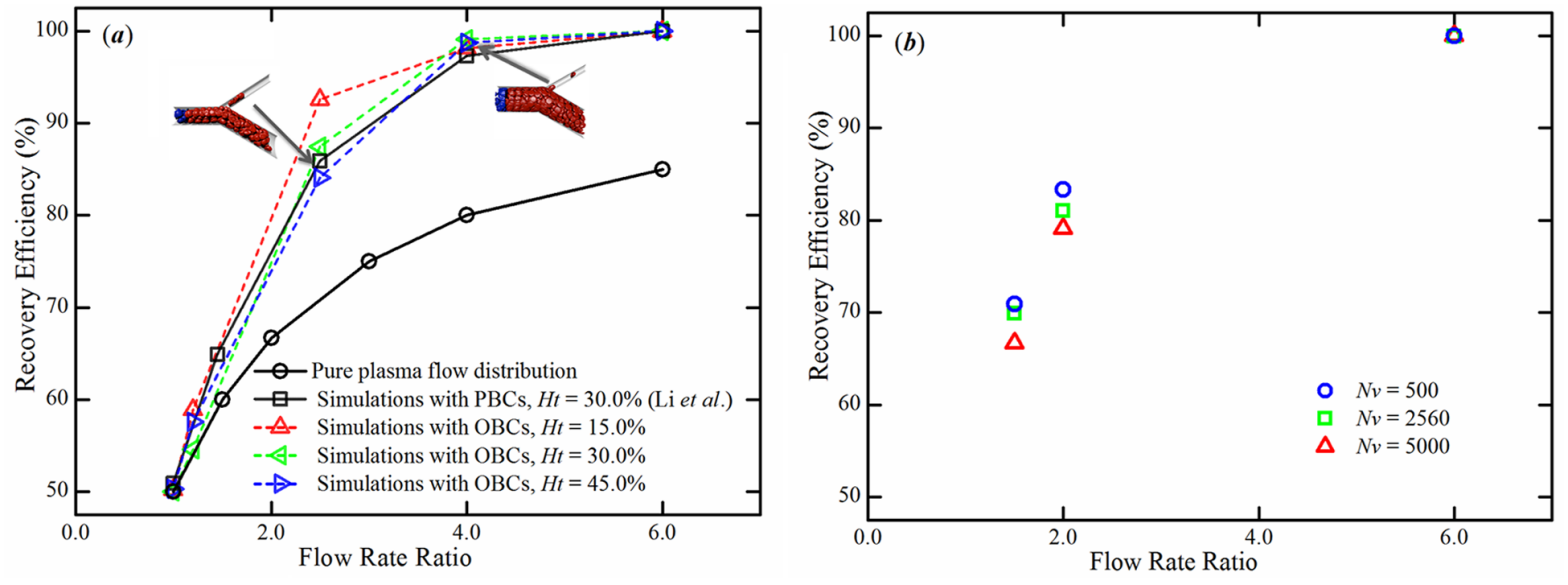
Particle recovery efficiency with respect to flow rate ratio. (*a*) Particle recovery efficiency at different hematocrit levels. Two snapshots of the RBCs at microvascular bifurcations with flow rate ratios of 2.5 and 4.0 at *H*_*t*_ = 45.0% are shown. Simulation data (black squares) from [32] are shown. (*b*) Particle recovery efficiency at different levels of coarse-graining of the MS-RBC model at *H*_*t*_ = 15.0%. The simulations are conducted using the MS-RBC model with *N*_*v*_ = 500, 2560 and 5000.

Next, we apply the proposed OBCs to model the blood flow in microvascular bifurcations. We used flow rate ratios, *ϕ*_*d*_, between branches 1.0, 1.2, 2.5, 4.0 and 6.0 and studied the particle recovery efficiency (the proportion of parent RBC flux entering each daughter branch) with respect to the flow rate ratios between two daughter branches. We perform simulations with three hematocrit levels (*H*_*t*_ = 15.0%, 30.0% and 45.0%) and find that the higher flow rate ratio yields the higher particle recovery efficiency. A 100% recovery efficiency is achieved for the flow rate ratios starting at 6:1. We also demonstrate that the higher the hematocrit is the higher the probability for a RBC is to follow a lower flow rate branch (see Fig 5***a***). This trend is similar to our previous observation from simulations of blood flow with the PBCs [32]. We also simulate the blood flow and study the particle recovery efficiency at different levels of coarse graining with *N*_*v*_ = 500, 2560 and 5000, using the MS-RBC model, see Fig 5***b***. We find that the particle recovery efficiency value increases somewhat with finer DPD resolution. For example, for *ϕ*_*d*_ = 1.5, the value of particle recovery efficiency shifts from 66.7% with *N*_*v*_ = 500 to 69.9% with *N*_*v*_ = 2560 and further to 70.9% with *N*_*v*_ = 5000; for *ϕ*_*d*_ = 2.0, it rises from 79.1% with *N*_*v*_ = 500 up to 81.0% with *N*_*v*_ = 2560 and then to 83.3% with *N*_*v*_ = 5000. At *ϕ*_*d*_ = 6:1, we find that 100% recovery efficiency can be achieved for all three of these cases.

**Fig 6:**
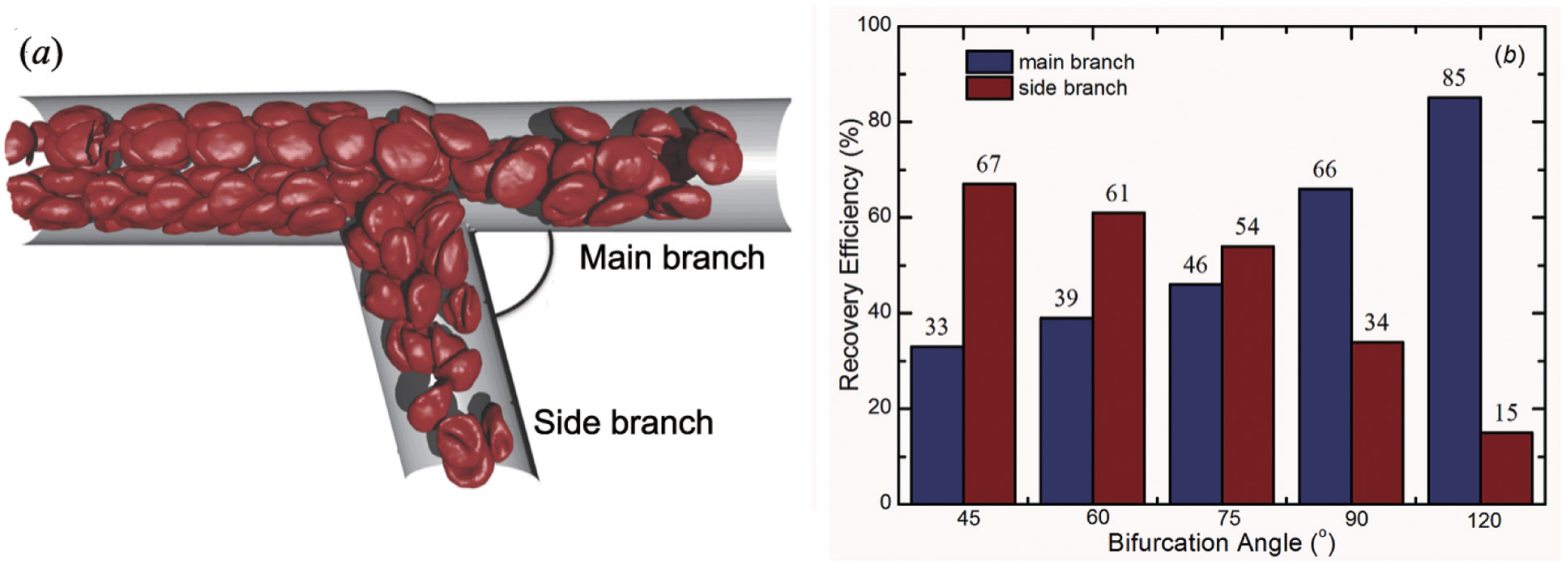
Effect of bifurcation angle on particle recovery efficiency. (***a***) A sketch of the microvascular bifurcation model by changing the bifurcation angle *θ*. In this model, the diameter of the parent branch is 20.0 *μm*, and the diameters of two daughter branches are both 16.5 *μm*. The average velocity of blood flow in parent branch is about 0.12 *mm*/*s*. (***b***) Relationship between the particle recovery efficiency and bifurcation angle.

The blood flow and stress characteristics in human arteriolar bifurcations are affected by the branch location and bifurcation angle variation [47, 48]. The proposed approach offers an effective method in investigating these effects on the behavior of RBCs flowing through a microvascular bifurcation. We then control the flow rate ratio by changing the bifurcation angle, *θ*, between the two daughter branches (see Fig 6***a***) at a fixed flow rate in the parent branch. We find that the particle recovery efficiency is clearly different in the small angle and in the large angle bifurcation, see Fig 6***b***. More RBCs move into the side branch at a smaller angle bifurcation, while more RBCs move into the main branch at a larger *θ*. A critical angle value can be estimated at *θ* ≈ 78^*o*^ when nearly half of the RBCs move into each branch. Therefore, our 3D DPD simulations demonstrated that the bifurcation angle influences the RBC flux to the daughter branches so that its effect on the RBC flux distributions in microvascular bifurcation cannot be neglected.

**Fig 7:**
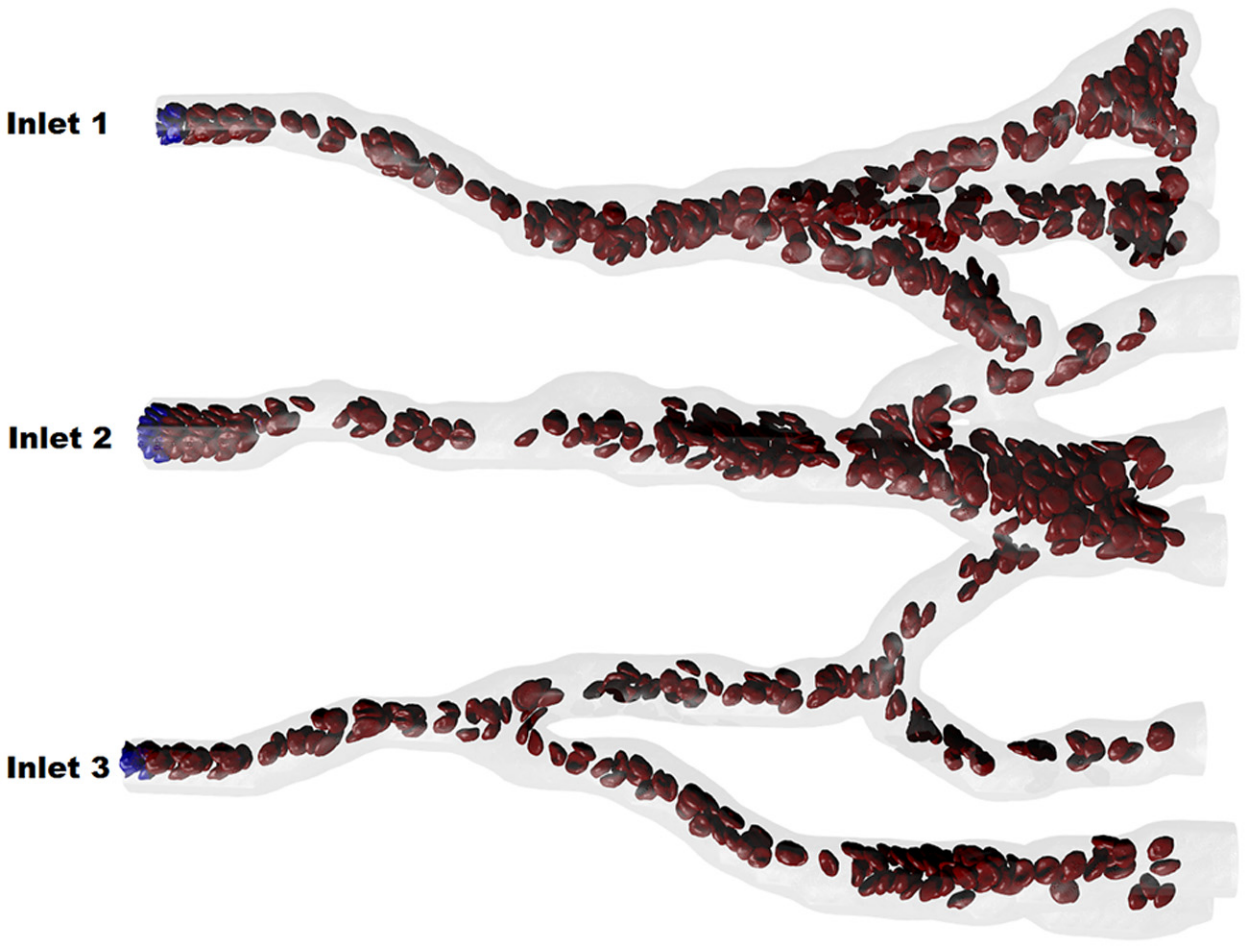
Snaphot for simulation of the blood flow in part of arterial network with three inlets and multiple outlets. The complex microvascular network was constructed using an angiogenesis model [49]. The walls of the microvascular network were formed by stationary DPD particles. An extra bounce-back reflection was applied to fluid particles to prevent them from entering the solid wall domain.

Finally, we demonstrate that the proposed approach can be employed to target blood flow simulations for multiple inlets and outlets. Here, for illustration purposes, we present a simulation of blood flow in a complex arterial network, which was constructed using the angiogenesis model [49]. As shown in Fig 7 and the S1 Video, the network has three inlets and multiple outlets. An interesting observation is that the number of fluid particles and the number of RBCs are almost constant during the simulations. Thus, we can simulate arterial blood flow and study the effect of combined different outflow and Dirichlet boundaries on the flow pattern.

In summary, in this paper, we have developed a general parallel framework for open boundary conditions including the inflow and outflow boundaries for particle-based methods. The approach presented above offers a straightforward way for an open system simulations such as blood flow in arteriolar bifurcations, which provides the possibility to simulate particulate flows for various systems with open boundaries. It was implemented as an extension to the Large-scale Atomic/Molecular Massively Parallel Simulator (LAMMPS) [50] and extensively tested in simulations for different domains in the High Performance Computing environment. To the best of our knowledge, this is the first simulation of blood flow in an arterial network with the MS-RBC model. Due to the nature of the proposed technique, there are some disadvantages to consider. First, the inflow at the inlet is duplicated from a fully developed flow generated in a pilot simulation with PBCs, thus, the proposed technique can not be used if the recirculation region is present at the inflow. Second, as the implementation of the proposed technique requires significant increase in communication among computational nodes in comparison to that of normal PBC systems. It is efficient for flow of bodies whose size is much smaller than that of the computational subdomains assigned to a process; however, it may be inappropriate when modeling an immersed body with a long chain structure (such as long polymer chains) due to overhead on the communication when generating and removing the immersed body.

## Supporting Information

S1 Video Simulation of blood flow at *H*_*t*_ = 15.0% in a 3D arterial network. The arterial network has three inlets and multiple outlets, and each of them has an internal diameter ranged from 28.0 *μm* to 40.0 *μm*. The network is modeled in a simulation box of size 192.0 *μm* × 250.0 *μm* × 311.0 *μm* with a total of 3,254,000 plasma particles in the system.
(GIF)
