# Supplementary figures and images for: Inflow/Outflow Boundary Conditions for Particle-Based Blood Flow Simulations: Application to Arterial Bifurcations and Trees
Source: PLoS Comput Biol. 2015 Aug 28;11(8):e1004410. doi: 10.1371/journal.pcbi.1004410 (PMC4552763; doi:10.1371/journal.pcbi.1004410)

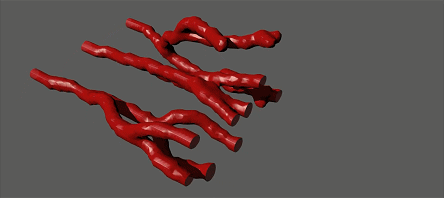

Supplement: S1 Video — The arterial network has three inlets and multiple outlets, and each of them has an internal diameter ranged from 28.0 μm to 40.0 μm. The network is modeled in a simulation box of size 192.0 μm × 250.0 μm × 311.0 μm with a total of 3,254,000 plasma particles in the system. (GIF) [file pcbi.1004410.s001.gif]
